# Expression Profile of Diabetes-Related Genes Associated with Leukocyte Sirtuin 1 Overexpression in Gestational Diabetes

**DOI:** 10.3390/ijms19123826

**Published:** 2018-11-30

**Authors:** Katarzyna Mac-Marcjanek, Andrzej Zieleniak, Monika Zurawska-Klis, Katarzyna Cypryk, Lucyna Wozniak, Marzena Wojcik

**Affiliations:** 1 Department of Structural Biology, Faculty of Biomedical Sciences and Postgraduate Education, Medical University of Lodz, 90-752 Lodz, Poland; k.mac.marcjanek@gmail.com (K.M.-M.); andrzej.zieleniak@umed.lodz.pl (A.Z.); 2 Diabetology and Metabolic Diseases Department, Medical University of Lodz, 92-213 Lodz; monika.zurawska-klis@umed.lodz.pl (M.Z.-K.); katarzyna.cypryk@umed.lodz.pl (K.C.)

**Keywords:** gene expression, gestational diabetes mellitus (GDM), Ingenuity Pathway Analysis (IPA), PCR array, Sirtuin 1 (SIRT1)

## Abstract

Although compelling evidence indicates that Sirtuin 1 (SIRT1) plays a prominent role in type 2 diabetes, its relationship with gestational diabetes (GDM) remains elusive. This study was aimed at identifying diabetes-related genes and cellular pathways linked to changes of leukocyte *SIRT1* expression at the time of GDM diagnosis. For this purpose, 122 GDM patients were screened for leukocyte *SIRT1* expression, and two subgroups were distinguished, namely GDM/*SIRT1*(↑) (*n* = 30, *p* < 0.05) and GDM/*SIRT1*(↔) (*n* = 92, *p* > 0.05), with significant and insignificant changes in leukocyte *SIRT1* expression compared to a normal glucose tolerant (NGT) group (*n* = 41), respectively. PCR array analysis identified 11 diabetes-related genes with at least a ± 2-fold difference in expression in GDM/*SIRT1*(↑) patients (*n* = 9) vs. NGT controls (*n* = 7); in addition, significant differences in the expression of four of the six investigated genes were confirmed between the entire GDM/*SIRT1*(↑) group and the whole NGT group (*p* < 0.05). Interestingly, of these four genes, only *ACLY* expression was found to significantly differ between GDM/*SIRT1*(↑) and GDM/*SIRT1*(↔). This study demonstrates that under hyperglycemic conditions, leukocyte *SIRT1* overexpression is accompanied by an over-abundance of three transcripts and an under-abundance of another; these four govern related metabolism, inflammation, and transport functions, suggesting that such alterations might represent systemic biological adaptations with a unique *ACLY* under-expression in GDM/*SIRT1*(↑) women.

## 1. Introduction

Gestational diabetes mellitus (GDM), defined as a carbohydrate intolerance of varying severity with onset or first recognition during pregnancy, is one of the most common pregnancy complications, affecting between 3% and 17% of all pregnancies, depending on the population studied and the diagnostic criteria used [1]. Its occurrence is associated with an increased risk of adverse maternal and perinatal outcomes, including preeclampsia, preterm delivery, Cesarean section, macrosomia, and respiratory distress syndrome [2]. Additionally, patients with GDM are more prone to developing type 2 diabetes mellitus (T2DM) and cardiovascular disease later in life than non-diabetic subjects and their offspring are also at an increased risk of developing obesity, metabolic syndrome, and T2DM during childhood and adolescence [3,4]. 

GDM and T2DM share some common features underlying pathophysiology. Both disorders are characterized by peripheral insulin resistance combined with a relative insufficiency in pancreatic beta-cell insulin production. Although there is no consensus in the pathophysiology of GDM, an altered adipokine metabolism, pro-inflammatory state, and exaggerated oxidative stress have been identified as important contributors [5,6,7,8].

Silent mating type information regulation 2 homolog 1 (SIRT1), a nicotine adenine dinucleotide (NAD+)-dependent class III protein deacetylase, is currently considered as a powerful regulator of cellular metabolism. It is believed to act as a pivotal mediator in coordinating metabolic responses to nutritional availability; hence, changes in its expression are believed to be associated with many pathological conditions, with diabetes being one of them.

It has been well-documented that SIRT1 increases insulin sensitivity in major insulin sensitive tissues, such as liver, skeletal muscle, and adipose tissue, and induces insulin secretion by pancreatic beta-cells [9,10]. Moreover, several studies have shown that Sirt1 overexpression protects animals against high fat diet (HFD)-induced glucose resistance [11,12], whereas SIRT1 down-regulation is associated with impaired glucose tolerance in individuals with metabolic syndrome and T2DM [13,14]. Similarly, pharmacological stimulation of SIRT1 by sirtuin-activating compounds (STACs), such as resveratrol, SRT1720, and SRT2104, has been reported to protect against HFD-induced obesity and insulin resistance in animals and humans [15,16,17,18]. However, it remains uncertain whether resveratrol and SRT1720 may activate SIRT1 directly [19]. It is noteworthy that despite these promising results, it has been found that patients with well-controlled T2DM who received 150 mg/day resveratrol for 30 days failed to demonstrate any improvement in hepatic or peripheral insulin sensitivity [20]. In addition, some reports have revealed decreased blood glucose levels in Sirt1 knockout mice compared to wild-type mice [21], and increased glucose production by cultured rat hepatoma cells after treatment with resveratrol [22].

Despite the fact that SIRT1 is increasingly being recognized for its growing number of biological roles in T2DM, much remains to be learned about the pathophysiologic implications of SIRT1-dependent alterations in GDM. To date, no systematic studies of SIRT1 expression under diabetic pregnancy have been performed, although some reports in this field have demonstrated disease-related alterations in the SIRT1 level. For example, GDM and in vitro hyperglycemia were found to decrease SIRT1 production in fetal endothelial colony-forming cells (ECFCs) and human umbilical vein endothelial cells (HUVECs) [23], while resveratrol was shown to impair GDM symptoms, such as hyperglycemia and insulin resistance, in the *db*/+ genetic GDM mouse model [24]. On the other hand, a more recent study showed increased *SIRT1* expression at one day postpartum in GDM women as a consequence of their exposure to hyperglycemia during GDM [25].

The present study examines GDM-induced molecular changes associated with increased *SIRT1* mRNA expression in leukocytes at the time of GDM diagnosis. In the initial stage, GDM patients were screened for leukocyte *SIRT1* expression. Since GDM and T2DM share an underlying pathophysiology, suggesting that a striking parallel may exist between these two diseases on the genetic level, the following stages went on to investigate specific changes in diabetes-relevant genes in the leukocytes of GDM patients with *SIRT1* overexpression. These analyses were performed using Reverse Transcriptase-quantitative Polymerase Chain Reaction (RT-qPCR) array technology: a powerful molecular tool used for simultaneously investigating gene expression in various tissues, including the blood [26]. To explore possible mechanisms of action for the identified gene candidates, an analysis was performed of gene networks and pathways by Ingenuity Pathway Analysis (IPA). Peripheral blood leukocytes were chosen for analyzing the expression of *SIRT1* and diabetes-related genes in pregnancies with GDM and those with normal glucose tolerance (NGT) since these cells are able to reflect pathological changes occurring in different tissues of the body: their use has been recommended in exploratory studies where metabolic tissues are not available, as in the case of pregnant women [27].

## 2. Results

### 2.1. Differential Clinical Characteristics and Leukocyte SIRT1 Gene Expression in the Studied Groups

The clinical data and leukocyte *SIRT1* expression data for different experimental groups are listed in Table 1. The univariate correlations between the leukocyte *SIRT1* mRNA and baseline characteristics of the subgroups studied are presented in Table 2.

#### 2.1.1. GDM Patients Versus Control Subjects

As expected, women with GDM (*n* = 122) had higher FPG, 1-h, and 2-h plasma glucose concentrations during the OGTT (*p* < 0.001) compared to NGT controls (*n* = 41) (Table 1). No significant difference was observed between the groups with respect to maternal age, degree of whole adiposity (i.e., the indices of pre-pregnancy and pregnancy BMI as well as body weight gain), lipid profile, (i.e., TGs, LDL-C, HDL-C, and TC), inflammatory marker (CRP) or insulin-resistance indices, such as plasma insulin level and HOMA-IR (*p* > 0.05). Interestingly, PCR analyses indicated that the leukocyte *SIRT1* mRNA level was significantly higher in the hyperglycemic GDM group compared to the NGT group (1.32-fold difference between the groups; *p =* 0.041), even after adjusting for age and obesity parameters, such as pre- and pregnancy BMI and gestational body weight gain (Table 3). In leukocyte, *SIRT1* gene expression showed a significant positive correlation with the 2-h post-load glucose concentration (*R* = 0.23, *p =* 0.005) in the entire study group (GDM+NGT, *n* = 163). No other correlations were found between leukocyte *SIRT1* mRNA and clinical variables (Table 2).

#### 2.1.2. GDM/*SIRT1*(↑) Patients Versus Control Subjects

The GDM group (*n* = 122) was stratified into the two subgroups, GDM/*SIRT1*(↑) (*n* = 30) and GDM/*SIRT1*(↔) (*n* = 92), based on the cut-off value for leukocyte *SIRT1* mRNA given in Materials and Methods. The results of the Kruskal-Wallis test identified a significant difference between the three groups with regard to fasting and post-load glucose (*p* < 0.001): GDM/*SIRT1*(↑), GDM/*SIRT1*(↔), and NGT (*p* < 0.05). The pairwise post hoc test for multiple comparisons revealed significantly higher fasting and post-load glucose concentrations in the two GDM subgroups compared to the NGT controls (*p* < 0.05). Although the values of these parameters were higher in the GDM/*SIRT1*(↑) group than the GDM/*SIRT1*(↔) group, they did not reach statistical significance (*p* > 0.05) (Table 1).

**Figure 1:**
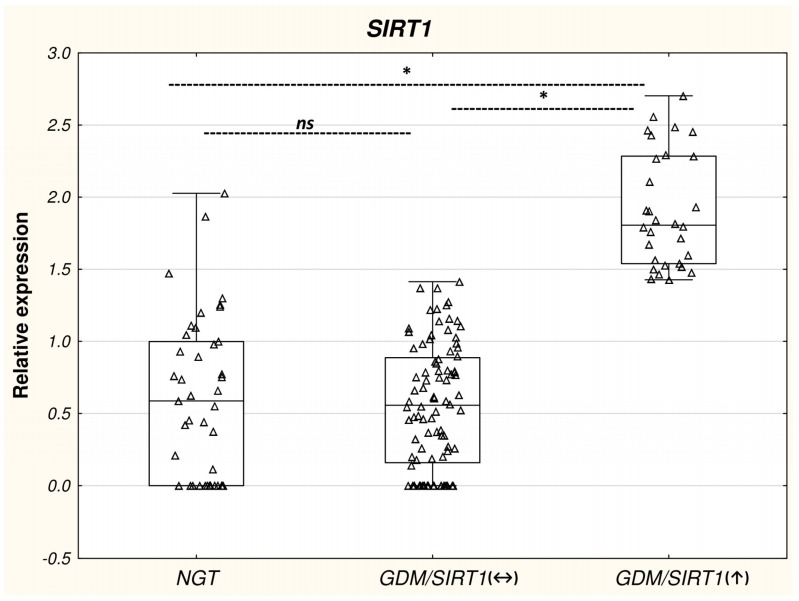
Comparison of leukocyte *SIRT1* mRNA expression in the NGT (*n* = 41), GDM/*SIRT1*(↑) (*n* = 30) and GDM/*SIRT1*(↔) (*n* = 92) groups. Leukocyte *SIRT1* mRNA level was normalized to a mean of the endogenous control *GAPDH*. Data are expressed as median ± interquartile range (25–75%), * *p* < 0.05 as assessed by post hoc test for pairwise multiple comparisons of mean rank sums; n.s.: non-significant.

As expected, the leukocyte *SIRT1* expression was significantly higher in the GDM/*SIRT1*(↑) group than the GDM/*SIRT1*(↔) and NGT groups (*p* < 0.05), with a 3.23- and 3.07-fold up-regulation, respectively; however, the level was comparable between the GDM/*SIRT1*(↔) and NGT groups (*p* > 0.05) (Table 1, Figure 1).

The univariate correlation analyses revealed significant positive associations between leukocyte *SIRT1* gene expression and plasma glucose measurements at fasting (*R* = 0.41, *p <* 0.001) and when following the OGTT (*R* = 0.32, *p =* 0.015 and *R* = 0.76, *p <* 0.001 for 1-h and 2-h post-load glucose concentrations, respectively), as well as with plasma HbA1c level (*R* = 0.31, *p =* 0.010) in the entire study group (NGT+ GDM/*SIRT1*(↑), *n* = 71) (Table 2).

### 2.2. Differential mRNA Expression of Diabetes-Related Genes in Leukocytes from GDM/SIRT1(↑) Patients Versus NGT Controls

To identify a transcriptional signature associated with leukocyte *SIRT1* overexpression, the Human Diabetes RT² Profiler™ PCR Array was used to compare the expression profile of diabetes-related genes in leukocytes from hyperglycemic GDM/*SIRT1*(↑) patients (*n* = 9) with that of the NGT subjects (*n* = 7). As shown in Table S1, out of 84 diabetes-related genes broadly categorized into the six functional clusters according to the Qiagen list, as detailed in the Materials and Methods section, 68 (80.1%) were detectable (Ct < 35) via the PCR profiler array in leukocytes. Among these transcripts, four were up-regulated (*SNAP23*, *G6PD*, *IL6*, and *IRS2*) and seven were down-regulated (*ACLY*, *GPD1*, *NSF*, *STXBP2*, *PDX1*, *SREBF1*, and *IRS1*), with a two-fold or greater expression difference between the GDM/*SIRT1*(↑) and NGT groups (Table 4). Out of the 11 differentially expressed genes in the GDM/*SIRT1*(↑) subjects, those encoding metabolic enzymes (*ACLY*, *G6PD*, *GPD1*) and molecules involved in receptor and molecular transport functions (*NSF*, *SNAP23*, *STXBP2*) represent the two dominant functional clusters (54.5%). Other abnormally expressed genes were found to encode the transcription factors *PDX1* and *SREBF1*, which are engaged in the regulation of insulin gene expression and lipid metabolism, respectively, as well as insulin receptor substrates *IRS1* and *IRS2* and pro-inflammatory cytokine *IL6*.

### 2.3. Real-time PCR Verification of the Selected Genes Among Study Groups

**Figure 2:**
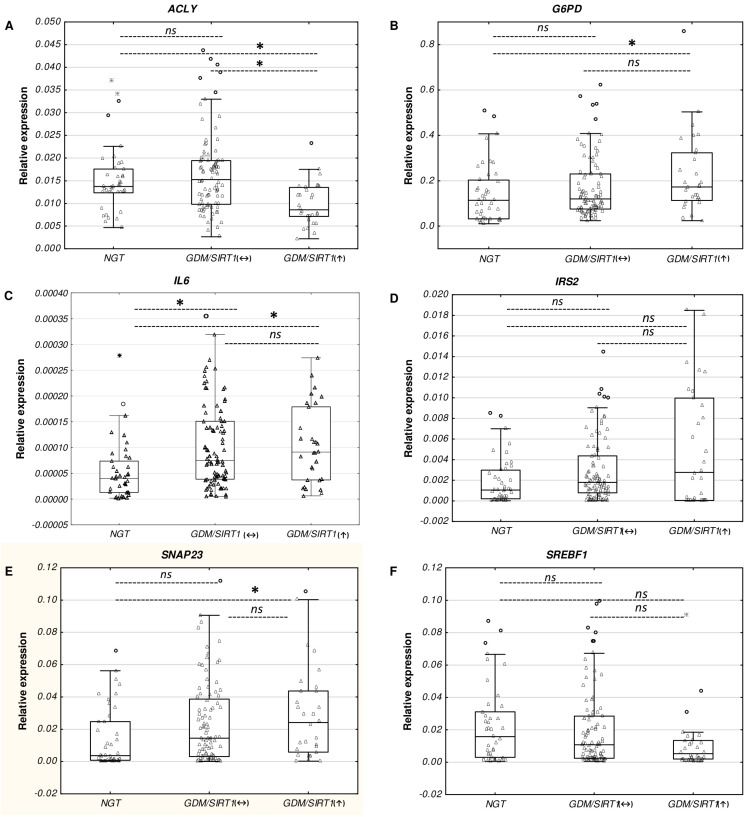
Verification results of the genes *ACLY* (**A**), *G6PD* (**B**), *IL6* (**C**), *IRS2* (**D**), *SNAP23* (**E**), and *SREBF1* (**F**) in NGT (*n* = 41), GDM/*SIRT1*(↔) (*n* = 92), and GDM/*SIRT1*(↑) (*n* = 30) groups by RT-qPCR. All mRNA levels of the investigated genes were normalized to a mean of the endogenous control *GAPDH*. Data are expressed as median ± interquartile range (25–75%); * *p* < 0.05, as assessed by post hoc test for pairwise multiple comparisons of mean rank sums; n.s.: non-significant.

The next stage examined whether gene expression changes for the six selected genes with an FC of <−2 or >2 (*SNAP23*, *G6PD*, *IL6*, *IRS2*, *ACLY*, *SREBF1*) in the pooled RNA samples of the GDM/*SIRT1*(↑) patients in a PCR profiler array could be representative for this group in general. To achieve this, RT-qPCR was used to compare the expression levels of the six gene transcripts in the three groups: RNA samples (*n* = 30) from GDM/*SIRT1*(↑) individuals versus RNA samples (*n* = 90) from GDM/*SIRT1*(↔) patients versus RNA samples (*n* = 41) from NGT subjects (Table 5 and Figure 2). The selected genes represented each of the five functional categories, including receptors and transporters (*SNAP23*), metabolic enzymes (*ACLY*, *G6PD*), secreted factors (*IL6*), signal transduction molecules (*IRS2*), and transcription factors (*SREBF1*). To make the findings more robust, the analysis employed different primers sets from those used in the PCR array (Table 8 in Materials and Methods). The gene expression analysis revealed that of the six selected genes, four demonstrated a significantly different expression between the GDM/*SIRT1*(↑) and the NGT group (*p <* 0.05): *ACLY* (FC = 0.63) displayed a significantly lower expression, while *G6PD* (FC = 1.52), *IL6* (FC = 2.29), and *SNAP23* (FC = 6.55) demonstrated a significantly increased expression. The other two genes, *IRS2* (FC = 2.62) and *SREBF1* (FC = 0.34), were up-regulated and down-regulated in the GDM/*SIRT1*(↑) group, respectively; however, these differences were not statistically significant (*p* > 0.05, Table 5). Importantly, the genes were found to be regulated in the same direction in both the pooled RNA (PCR array) and non-pooled RNA (RT-qPCR verification) samples of the GDM/*SIRT1*(↑) patients. It is noteworthy that the FCs observed for four of the genes (*ACLY*, *IL6*, *IRS2*, *SREBF1*) were close to those obtained from the PCR array, whereas the FCs for *G6PD* and *SNAP23* were approximately two times lower and higher, respectively, than those obtained from the PCR array.

When the expression of the six selected genes was compared between the GDM/*SIRT1*(↔) and NGT groups, only *IL6* was significantly increased in the GDM/*SIRT1*(↔) group (FC = 1.88; *p* < 0.05); however, this increase was smaller than in the GDM/*SIRT1*(↑) group (FC = 2.29; *p* < 0.05). Of note, except the *ACLY* gene, all other genes were found to be regulated in the same direction as in the GDM/*SIRT1*(↑) group (Table 5 and Figure 2).

### 2.4. Functional analysis using Ingenuity Pathway Analysis (IPA)

**Figure 3:**
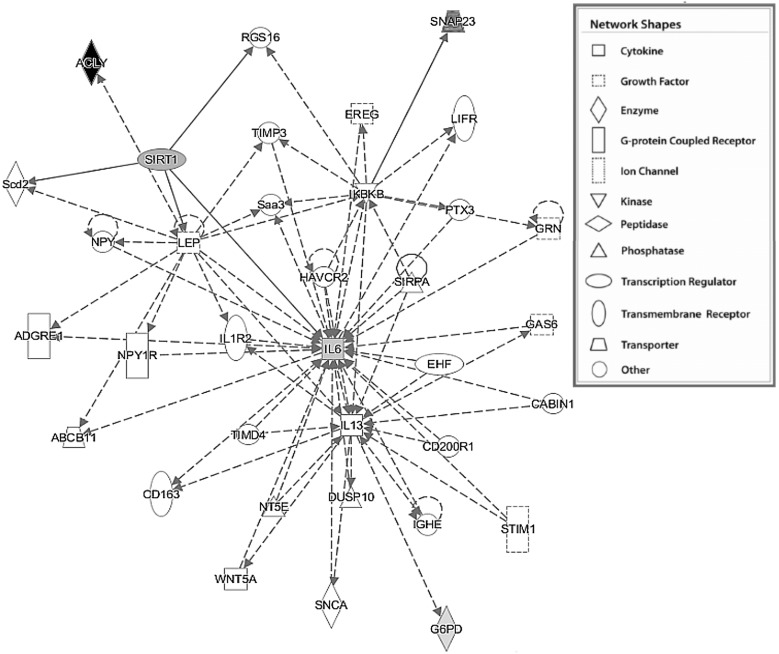
Cell-to cell signaling and interaction, hematological system development and function, inflammatory response network in the GDM/*SIRT1*(↑) patients, as identified by IPA. The five genes (*SIRT1*, *ACLY*, *G6PD*, *IL6*, and *SNAP23*) verified as significant in the entire GDM/*SIRT1*(↑) group (*p* < 0.05) were converted into a gene network by IPA software. The figure displays the network graphically as nodes (gene/gene product) and edges (biological relationship between nodes). The up-regulated genes are shown in gray shading, with the color intensity indicating the fold change in expression, whereas the down-regulated gene (*ACLY*) is shown in black. A direct relationship is presented as a straight line, and an indirect relationship as a dotted line. Genes involved in the network that are not included in the transcriptome results are shown in white. The node shapes are indicative of function, with the legend given to the right.

To further determine the biological significance and functional classification of the genes whose expression was statistically different in the GDM/*SIRT1*(↑) group (i.e., *SIRT1*, *ACLY*, *G6PD*, *IL6*, and *SNAP23*, *p <* 0.05), IPA analysis was performed. The most significant disorders of biological functions associated with these genes were found to be inflammatory response, cardiovascular disease, organismal injury and abnormalities, immunological disease, and inflammatory disease (Table 6). Cell-to cell signaling and interaction, cellular development, drug metabolism, molecular transport, and small molecule biochemistry were the most significant molecular and cellular functional categories. Hematological system development and function, immune cell trafficking, cardiovascular system development and function, organ morphology, and organismal development were the most significant categories in physiological development and system function (Table 6). The dominant canonical pathways included the Sirtuin signaling pathway, acetyl-CoA biosynthesis III (from citrate), the pentose phosphate pathway, and differential regulation of cytokine production in macrophage and T helper cells by IL17A and IL17F (Table 7). As shown in Figure 3, there was only one significant gene network involved in cell-to-cell signaling and interaction, hematological system development and function, inflammatory response (score 12) found in the GDM/*SIRT1*(↑) patients.

## 3. Discussion

GDM is a chronic, multifactorial, and complex disease involving transcriptional dysregulation in multiple tissues and organs, including leukocytes. As SIRT1 regulates a wide spectrum of important cellular processes, it is clear that changes in its expression may disturb many vital functions in the human body. Although a considerable body of evidence exists indicating that SIRT1 may play a role in T2DM, there is actually no consensus as to whether it is relevant to GDM. To clarify this issue, this unique and multistage study systematically examines the molecular changes associated with *SIRT1* overexpression in leukocytes from a group of clinically well-characterized diabetic pregnancies displaying homogeneous leukocyte *SIRT1* expression (i.e., the GDM/*SIRT1*(↑) group) at the time of GDM diagnosis.

In the first stage of the study, the leukocyte *SIRT1* mRNA level was found to be significantly higher in the entire GDM (*n* = 122) than the NGT (*n* = 41) groups, which also correlated with glucose metabolism; this relationship suggests that a close link may exist between leukocyte *SIRT1* expression and glucose homeostasis. This finding was unexpected, especially in view of previous data showing decreased SIRT1 expression or activity in patients with T2DM or metabolic syndrome [13,28]. Although the reason for this discrepancy is unclear, it may be due to the fact that previous studies were based on a different cell type: peripheral blood mononuclear cells (PBMC) were used by de Kreutzenberg, while whole leukocytes were used in the present study [13]. Significantly higher *SIRT1* mRNA expression has been reported in granulocytes compared with lymphocytes and monocytes [29]. Alternatively, the differences may be attributed to patient characteristics: firstly, the patients enrolled in the present study were younger (30.5 (27.0–33.0) years) than those included in previous studies (48.0 ± 1.5 years and 62.01 ± 8.67 years) and secondly, while the GDM and NGT groups in the present study demonstrated similar parameters of adiposity (i.e., pre-and pregnancy BMI, body weight gain) and insulin resistance (i.e., insulin level and HOMA-IR), the insulin resistant subjects in the de Kreutzenberg study [13] and the Song study [28] presented increased BMI. Nevertheless, our findings are in line with those of a more recent study revealing increased *SIRT1* expression at one day postpartum in GDM women as a consequence of their exposure to hyperglycemia during GDM [25]. Hence, our results suggest that increased leukocyte *SIRT1* mRNA expression plays a role in diabetic pregnancy; they also enrich the current understanding of the field of GDM biology, in that *SIRT1* overexpression appears to be important not only during the postpartum period following pregnancy complicated by GDM, but also at the time of GDM diagnosis itself. Low to moderate (2.5- to 7.5-fold) cardiac-specific *Sirt1* overexpression in transgenic mice has been demonstrated to protect cardiomyocytes from apoptosis and age-dependent degeneration, whereas its greater increase (12.5-fold) has a detrimental effect on these cells, leading to cardiomyopathy [30].

SIRT1 expression has been found to generally decrease with age and obesity [31,32]. However, since maternal age and adiposity were comparable between women with and without GDM in the present study, and furthermore, since leukocyte *SIRT1* expression remained significantly increased in the GDM group, even after adjustment for these potentially relevant variables, we may exclude their possible effect on leukocyte *SIRT1* up-regulation in GDM women.

Although calorie restriction (CR) has been proven to extend the lifespan in lower organisms and mammals through its effect on sirtuins, as evidenced by increased SIRT1 expression in the adipose tissue, liver, kidney, and brain tissue of rodents, as well as in PBMC [33,34,35], it has also been found that the plasma SIRT1 concentration falls in pregnant women during Ramadan fasting to a degree that correlates inversely with the number of fasting days [36]. Physical activity has also been documented as a positive regulator of SIRT1 expression in rats and humans [37,38]. Unfortunately, as no information was available on diet and excise interventions among the pregnancies enrolled in our study, it was not possible to consider their impact on leukocyte *SIRT1* expression in GDM patients. In addition, the 12-h overnight fast undertaken by the study participants before blood sampling in the present study appears to be too short to increase the *SIRT1* mRNA level in their leukocytes [13,39].

To further clarify the metabolic and transcriptional rearrangements associated with leukocyte *SIRT1* overexpression during GDM, a stratified expression analysis of leukocyte *SIRT1* status was performed in the GDM patients, which allowed a GDM/*SIRT1*(↑) (*n* = 30) group to be discriminated from a GDM/*SIRT1*(↔) (*n* = 92) group. Such molecular stratification of patients is a practical approach for better interpretation of experimental results and more effective characterization of the disease in cases when large diversity is present in the gene expression data obtained from clinical samples. Compared to the NGT group, the two GDM groups exhibited significantly higher fasting and post-load glucose concentrations, which, along with the plasma HbA1c level, positively correlated with leukocyte *SIRT1* expression; this implies a linkage between leukocyte *SIRT1* mRNA up-regulation and both the early and late phase of hyperglycemia. Of note, the glycemic measurements were increased in the GDM/*SIRT1*(↑) group compared to the GDM/*SIRT1*(↔) group, but this difference was not statistically significant.

As the complex molecular events related with *SIRT1* overexpression in leukocytes of GDM patients were unknown, the study also compared the changes in leukocyte-induced expression of diabetes-related genes taking place in hyperglycemic GDM/*SIRT1*(↑) women with those in the NGT controls using RT-qPCR array technology. Such an approach has previously led to the successful identification of key factors involved in T2DM development [40]. The present study used the pooled RNA samples of the GDM/*SIRT1*(↑) (*n* = 9) and the NGT (*n* = 7) patients to eliminate donor-donor variation and to obtain results reflecting consistent *SIRT1*-related transcriptomic changes in diabetes-related genes: a strategy previously described in other studies [41,42]. The current study identified an 11-gene signature that accurately distinguished the GDM/*SIRT1*(↑) group from the NGT group based on at least a two-fold difference in expression, with four genes up-regulated and seven down-regulated in the GDM group compared to the NGT group. These were assigned to five different functional clusters, including metabolic enzymes (*ACLY, G6PD, GPD1*), molecular transport functions *(NSF, SNAP23, STXBP2*), transcription factors (*PDX1* and *SREBF1*), signal transduction molecules (*IRS1* and *IRS2*), and an inflammatory factor (*IL6*). Thus, leukocyte *SIRT1* overexpression in the GDM/*SIRT1*(↑) patients is closely related to transcriptional changes in genes involved with a diverse set of biological functions, and the importance of several of them has been attributed to GDM-related functions [43,44].

Since quantitative and qualitative differences were seen in the transcriptional expression pattern of leukocytes between the pooled RNA samples of GDM/*SIRT1*(↑) and NGT women, the next stage more closely examined the PCR array data in the entire experimental groups of GDM/*SIRT1*(↑) (*n* = 30) versus NGT (*n* = 41). Of the 11 genes, six were selected which represented each of the five functional clusters: *SNAP23*, *ACLY, G6PD*, *IL6*, *IRS2*, and *SREBF1*. Comparative expression analysis of all these genes revealed significantly higher levels of *G6PD* (FC = 1.52)*, IL6* (FC = 2.28)*,* and *SNAP23* (FC = 6.55) transcripts and a lower level of the *ACLY* transcript (FC = 0.63) in the GDM/*SIRT1*(↑) group compared to the NGT group, whereas no significant differences were found between the groups with regard to *IRS2* (FC = 2.62) and *SREBF1* (FC = 0.34) expression. Nevertheless, the expression of all analyzed genes among the GDM/*SIRT1*(↑) group was regulated in the same direction as on the PCR array, confirming the reliability of the results obtained, irrespective of the RNAs used (pooled versus non-pooled) and different primer sets employed in the two methods. When the expression of the aforementioned genes was further analyzed between the GDM/*SIRT1*(↔) and NGT groups, only the expression of *IL6* (FC = 1.88) was significantly higher in the diabetic pregnancies; this suggests it is linked to GDM, regardless of the mRNA expression status of *SIRT1* in GDM patients.

IL6 is a pleiotropic cytokine that has been implicated not only in inflammation, but also in glucose metabolism, and its concentration in GDM patients has been found to be elevated, independent of BMI [45]. Although controversy still exists whether hyperglycemia is a cause or an effect of increased *IL6* expression [46,47,48], it is reasonable to assume that our finding of an increased leukocyte *IL6* expression in both groups of GDM patients may be associated with their hyperglycemic state. It is also possible that hyperglycemia-induced oxidative stress could participate in this event, since sugar-derived substances called advanced glycation end products (AGEs) have been reported to stimulate IL6 production in human monocytes [49]. Unfortunately, this study did not examine the oxidative stress markers that could fully confirm this hypothesis; further studies are clearly needed in this field since hyperglycemia-induced oxidative stress is an important contributor to GDM development, with health consequences for both mother and fetus [50].

Among the significantly up-regulated genes identified in the GDM/*SIRT1*(↑) patients, *SNAP23* was the most increased. The gene encodes synaptosomal-associated protein 23, a soluble *N*-ethyl-maleimide-sensitive fusion protein attachment protein receptor (SNARE) molecule, which is engaged in transport vesicle docking and fusion. Despite evidence indicating that a deficiency in several SNARE proteins is linked to T2DM development, and an increase of their cellular levels may lead to the maintenance of glucose homeostasis, the precise role of SNARE members in intracellular trafficking during diabetes is far from being well-understood [51]. Interestingly, a recent study by Rezaei Farimani et al. [52] reported that resveratrol increases *SNAP23* gene expression in diabetic animals; it is hence theoretically possible that the increased leukocyte *SNAP23* expression in the GDM/*SIRT1*(↑) group observed in our study could be an adaptive response to *SIRT1* overexpression in diabetic pregnancies. However, this remains speculative until specifically addressed in future studies.

Another gene of interest that showed a significant increase in expression in the GDM/*SIRT1*(↑) group was *G6PD*, encoding glucose-6-phosphate dehydrogenase (G6PD): the rate-limiting enzyme in the pentose-phosphate pathway that catalyzes the dehydrogenation reaction of glucose-6-phosphate to 6-phosphogluconolactone and reduces nicotinamide adenine dinucleotide phosphate (NADPH). NADPH is required not only for fatty acid and cholesterol biosynthesis, but also for the regeneration of reduced forms of the two important antioxidants, such as glutathione and thioredoxin. Hence, G6PD displays a cytoprotective action against oxidative stress, i.e., a key pathological factor associated with diabetes. Accumulating data indicates decreased G6PD activity related with oxidative damage, cellular dysfunction, and organ damage in endothelial cells, kidney, liver, and red blood cells under high glucose conditions [53,54,55]. On the other hand, increased G6PD activity has been shown to improve redox status and cell growth, and decrease cell death in endothelial cells [56]. Several studies also point to a potential link between G6PD deficiency and the development of diabetes, including GDM [57,58]. Recently, resveratrol has been found to improve G6PD activity in an animal model of diabetes [59]. These findings raise the possibility that the up-regulation of leukocyte *G6PD* observed in the GDM/*SIRT1*(↑) patients in the present study might be the adaptive mechanism to leukocyte *SIRT1* overexpression which would protect diabetic pregnancies against oxidative stress and cellular dysfunction. Of course, further studies are required to clarify whether leukocyte *SIRT1* and *G6PD* up-regulation might mediate antioxidative effects during gestational diabetes.

Out of the set of genes whose expression was found to be significantly changed in the GDM/*SIRT1*(↑) group, only *ACLY* was down-regulated. This gene encodes a cytoplasmic adenosine triphosphate (ATP) citrate lyase (ACL) that converts citrate to acetyl-coenzyme A (CoA), a key compound for de novo lipid synthesis. Hence, ACL is currently considered as a potential therapeutic target for lipid reduction in obesity and obesity-related metabolic diseases [60,61]. In addition, several observations support the notion that ACL is crucial for histone acetylation and the control of DNA accessibility for gene transcription [62]. In this regard, increased histone acetylation via the citrate lyase pathway has been shown in cultured mammalian cells under hyperglycemic conditions [63]. More speculatively, it seems logical that the leukocyte *ACLY* down-regulation observed in the GDM/*SIRT1*(↑) group in the present study might lead to impaired de novo lipogenesis; in addition, the GDM/*SIRT1*(↑) pregnancies also demonstrated lower leukocyte *SREBF1* expression than the NGT controls, although this change was not significant. The gene *SREBF1* encodes sterol regulatory element binding protein 1, which is a well-known transcriptional factor participating in the regulation of lipid metabolism by promoting the expression of numerous lipogenic genes, including *ACLY* [64]. On the other hand, it cannot be excluded that *ACLY* down-regulation might serve as the adaptive change to leukocyte *SIRT1* overexpression taking place in the GDM group, since SIRT1, acting as histone deacetylase, might diminish histone acetylation by inactivating histone acetyltransferase activity. Hence, it will be of great importance to investigate whether specific genes might be altered in leukocytes with increased *SIRT1* expression as a result of ACL inhibition. It is noteworthy that the GDM/*SIRT1*(↑) and GDM/*SIRT1*(↔) groups differed with regard to the directionality of leukocyte *ACLY* expression; as it was previously mentioned, the leukocyte *ACLY* mRNA level was significantly down-regulated in the GDM/*SIRT1*(↑) patients compared to both the NGT and GDM/*SIRT1*(↔) groups and non-significantly up-regulated in the GDM/*SIRT1*(↔) subjects compared to NGT controls, indicating that the effect of *ACLY* expression may be distinct in leukocytes, depending on the *SIRT1* expression status in these cells. Hence, leukocyte *ACLY* down-regulation in the GDM/*SIRT1*(↑) pregnancies at the time of GDM diagnosis appears to be specific in these subjects, making this gene potentially relevant for their metabolic responses.

In addition to identifying the individual genes linked to *SIRT1* overexpression, the study also analyzed the biological functions and networks in which the set of four significantly changed genes (*ACLY, G6PD, IL6, SNAP23*), along with *SIRT1*, may play critical roles. IPA was used to identify significant canonical pathways in leukocytes, including the Sirtuin signaling pathway, acetyl-CoA biosynthesis III (from citrate), and the pentose phosphate pathway; in addition, differential regulation of cytokine production was also identified in macrophage and T helper cells by IL17A and IL17F. According to the above described biological functions of the altered genes, their involvement in the first three pathways is not surprising. The latter pathway is involved in pathogenic events taking place during inflammatory diseases, since a pro-inflammatory cytokine IL17A has been shown to regulate NF-kappa B and mitogen-activated protein kinases (MAPK) and induces the expression of IL6 and cyclooxygenase 2 (COX2) [65,66]. A recent study of IL17 revealed that SIRT1 plays a protective role in proliferative diabetic retinopathy by suppressing IL17 production [67]. Among the most relevant biological functions of the aforementioned genes were inflammatory response, cardiovascular disease, cell-to cell signaling and interaction, cellular development, hematological system development and function, and immune cell trafficking, confirming their effect on changes in cellular and immunological functions in GDM [68,69]. Our analysis strategy also revealed the top biological gene interaction network containing the greatest number of differentially-expressed genes involved in cell-to-cell signaling and interaction, hematological system development and function, and the inflammatory response, thus highlighting the functional and biological importance of the set of selected genes in GDM.

The present study design has several limitations. First, it did not assess the expression of both *SIRT1* and selected genes at the protein level among all study participants. Therefore, further proteomics studies are needed to determine whether the identified changes in gene expression truly reflect the changes in their protein amounts or activities. Such investigations are currently under way in our research group. A second limitation is that mRNA from whole leukocytes was used; hence, the leukocyte transcriptional alterations observed in our study cannot reflect cell-type specific changes in the leukocyte subpopulations. However, our intent was to examine the overall response of leukocytes to metabolic changes occurring in GDM. A final limitation is the lack of PCR array results for the GDM/*SIRT1*(↔) subjects; however, as transcriptional differences in most diabetes-related genes were found to be modest between the GDM/*SIRT1*(↑) and NGT groups, it may be expected that they will be smaller between the GDM/*SIRT1*(↔) and NGT groups. Therefore, our research strategy was based on the identification of genes with differential expression (2-fold or more) by PCR array expression profiling of leukocytes from the small number of GDM/*SIRT1*(↑) patients, followed by verification of their expression in a relatively large number of diabetic patients of the GDM/*SIRT1*(↑) and GDM/*SIRT1*(↔) groups, thus improving the accuracy and reliability of our results.

## 4. Materials and Methods

### 4.1. Study Population

A total of 163 pregnant Caucasian women (41 with NGT and 122 with GDM) were enrolled and studied at the Outpatient Diabetological Clinic “OmniMed” in Lodz, Poland. All pregnant women underwent a 75 g oral glucose tolerance test (OGTT) at 24–28 weeks’ gestation, or later if it was not possible during this period. GDM was diagnosed according to the Polish Diabetes Association (PDA) 2011 guidelines (modified WHO diagnostic criteria) [70], with the following threshold glucose levels: fasting ≥100 mg/dL (5.6 mmol/L), 1 h ≥180 mg/dL (10.0 mmol/L), or 2 h ≥140 mg/dL (7.8 mmol/L) (PDA 2011). The pregnant NGT women had a negative screen.

All patients were eligible to participate unless they had one or more exclusion criteria: age <18 and >40 years, family history of diabetes in first-degree relatives, GDM in previous pregnancy, the occurrence of any form of pre-pregnancy diabetes, the presence of concomitant systemic disease (chronic or acute or infectious), and taking insulin or any oral hypoglycemic medications. Neither GDM nor NGT subjects were controlled in terms of diet and exercise before the overnight fast.

All clinical investigations were conducted in accordance with the guidelines of The Declaration of Helsinki, and approved by the Ethical Committee of the Medical University of Lodz (No. RNN/153/09/KB, 21 April 2009). All patients gave informed consent before joining the study.

### 4.2. Anthropometric and Biochemical Data

Data concerning maternal age and pre-pregnancy weight were provided by participants. Pregnancy height and weight were measured at the OGTT visit using standardized procedures and calibrated equipment. Body mass index (BMI) was calculated by dividing the weight in kilograms by the height in meters squared. 

Blood samples were collected from the patients after a 12-h overnight fast. Biochemical assays for determining blood glucose, insulin, glycated hemoglobin (HbA1c), C-reactive protein (CRP), and lipid profile, i.e., total cholesterol (TC), triglycerides (TGs), and high-density lipoprotein (HDL)- and low-density lipoprotein (LDL)-cholesterol, were performed with standard laboratory methods as previously described [71,72].

Plasma glucose and insulin values were used to calculate a homeostasis model assessment of insulin resistance (HOMA-IR) as follows [73]:

HOMA-IR = [fasting insulin (μU/mL) × fasting glucose (mg/dL)/405.

### 4.3. Leukocytes Separation

Blood (10 mL) was collected from patients in EDTA-containing tubes. Leukocytes were isolated immediately after blood draws as previously described [71]. Briefly, patient blood samples were spun down at 3000 rpm for 10 min at 4 °C and the plasma supernatant was discarded. After adding red blood cell lysis buffer (0.5M NH_4_Cl, 10 mM KHCO_3_, 10 mM EDTA, pH 8.0) and 30 min incubation on ice, the samples were centrifuged at 4000 rpm for 10 min at 4 °C. The pellets containing leukocytes were washed twice with the phosphate-buffered saline (PBS).

### 4.4. RNA extraction and SIRT1 Gene Expression Assay

Total RNA was extracted from leukocytes using commercially-available acid-phenol reagent (Tri Reagent, Sigma-Aldrich, St. Louis, MO, US), according to the manufacturer’s instruction, and its quantity and quality were assessed with a LAMBDA 25 UV spectrophotometer (PerkinElmer, Chorley, UK) at UV260 and UV260/280, respectively. RNA (1 μg) was converted to cDNA using the RevertAid™ First Strand cDNA Synthesis Kits (Fermentas, Vilnius, Lithuania), according to the manufacturer′s instructions.

Leukocyte *SIRT1* gene expression was determined in the GDM (*n* = 122) and NGT (n = 41) groups by semi-quantitative PCR carried out in a TPersonal 48 Thermocycler (Biometra, Göttingen, Germany) using a reaction mix containing cDNA (1 µL), *Taq* DNA polymerase (0.6 unit), dNTPs (200 µM), 10× reaction buffer, and forward and reverse primers (1 µM) for *SIRT1*, with the sequences depicted in Table 8. The leukocyte *SIRT1* expression level was normalized to the housekeeping gene *GAPDH*, encoding the glyceraldehyde 3-phosphate dehydrogenase, since its expression was stable between sample groups. After an initial denaturation at 95 °C for three minutes, amplification was performed with denaturation at 95 °C for 25 s, annealing at 56 °C for 30 s, and extension at 72 °C for 30 s for 27 cycles, followed by at 72 °C for 10 min. Each PCR reaction was run in duplicate. The amplification products (544-bp and 514-bp for *SIRT1* and *GAPDH*, respectively) were analyzed on 1.2% agarose gels stained with ethidium bromide and quantified by densitometry with the Gelix One 220 program (Biostep GmbH, Jahnsdorf, Germany).

Due to the high degree of heterogeneity in leukocyte *SIRT1* expression among the entire GDM group, the GDM patients were subsequently stratified into two subgroups: (i) a GDM/*SIRT1*(↑) group consisting of GDM patients (*n* = 30) who exhibited significantly increased leukocyte *SIRT1* expression compared to the NGT group (*p* < 0.05); and (ii) a GDM/*SIRT1*(↔) group consisting of GDM patients (*n* = 92) who exhibited unchanged leukocyte *SIRT1* expression compared to the NGT group (*p* > 0.05). The threshold limit for GDM group separation was demarcated experimentally to reach the following criteria: (i) the lack of significant differences in leukocyte *SIRT1* expression between the GDM/*SIRT1*(↔) and NGT groups; (ii) the occurrence of a significant difference in leukocyte *SIRT1* expression between the GDM/*SIRT1*(↑) and NGT groups; and (iii) the similarity of leukocyte *SIRT1* expression among the NGT and GDM/*SIRT1*(↔) groups. In order to establish the threshold limit value for GDM group stratification into the GDM/*SIRT1*(↔) and GDM/*SIRT1*(↑) subgroups, the 2 ΔC(T) value of 1.413 (the upper quartile equals to the 75th percentile of the data) was calculated.

### 4.5. Human Diabetes RT² Profiler™ PCR Array

The relative expression of diabetes-related genes was determined in leukocytes of the selected GDM patients (*n* = 9) with the highest *SIRT1* expression among the GDM group, namely GDM/*SIRT1*(↑), versus NGT controls (*n* = 7) by using a 96-well RT2 Profiler™PCR Array. (PAHS-023Z–Human Diabetes PCR Array, SA BiosciencesTM, A Qiagen Company, USA) This profiles the expression of 84 genes involved in the development and progression of diabetes, as well as five housekeeping genes (beta-2-microglobulin, *B2M*); hypoxanthine phosphoribosyltransferase 1, *HPRT1*; ribosomal protein L13a, *RPL13A*; *GAPDH*; actin beta, *ACTB*) by real-time PCR using the SYBR Green detection method. It also contains controls for genomic DNA contamination, RNA quality, and general PCR performance. The 84 genes investigated were grouped into six functional categories based on the Qiagen listing as follows: (i) receptors, transporters, and channels; (ii) nuclear receptors; (iii) metabolic enzymes; (iv) secreted factors; (v) signal transduction molecules; and (vi) transcription factors (Table S1).

Equal amounts of total RNA from samples taken from nine GDM/*SIRT1*(↑) patients were pooled together (1 μg), and then reverse transcribed to form cDNA using the RT2 First Strand Kit (C-03/330401, SA Biosciences™, A QIAGEN Company, Venlo, The Netherlands), as described by the manufacturer. The same procedure was applied for seven NGT controls. The expression of the 84 diabetes-related genes was then evaluated by diluting the samples in RT2 Real-Time™ SYBR Green PCR master mix according to the supplier’s directions, which were then pipetted into 96-well PCR array plates. Real-time PCR was performed in technical duplicates using the 7900HT Fast Real-Time PCR System (Applied Biosystems, Foster City, CA, USA). PCR reaction conditions were as follows: 95 °C for 10 min followed by 40 cycles of 95 °C for 15 s, and 60 °C for 1 min. Quality controls included within the array plates confirmed a lack of DNA contamination, and that RNA quality and PCR performance were acceptable.

Raw data obtained from the real-time PCR was analyzed using an MS-Excel sheet from the manufacturer’s website (http://www.sabiosciences.com/pcrarraydataanalysis.php), and the threshold cycle (Ct) cutoff was set at 35 cycles. For these calculations, the mean expression of the five reference genes was used, since it did not differ between the GDM and NGT groups. Fold changes (FC) in average gene expression were expressed as the difference in expression of the individual diabetes-related gene from the GDM/*SIRT1*(↑) group compared to those of the NGT group. Genes were considered to be up-regulated or down-regulated if changes in expression levels were ≥ 2-fold or ≤ 2-fold, respectively.

### 4.6. Quantitative RT-PCR Verification

To verify the findings of gene transcript measurements performed on the pooled RNA samples of the patients, RT-qPCR analysis was performed for the same six selected genes with altered expression (i.e., *ACLY*, *G6PD*, *IL6*, *IRS2*, *SNAP23* and *SREBF1*) in each of the RNA samples obtained from leukocytes of individuals belonging to the GDM/*SIRT1*(↑) (*n* = 30), GDM/*SIRT1*(↔) (*n* = 92), and NGT (*n* = 41) groups. For this purpose, total RNA was isolated from leukocytes of each GDM/*SIRT1*(↑)/GDM/*SIRT1*(↔)/NGT patient and quantified as described above. cDNA was synthesized from 1 µg total RNA using the RevertAid™ First Strand cDNA Synthesis Kits (Fermentas, Vilnius, Lithuania), according to the manufacturer’s recommendations. PCR reactions were carried out in duplicate with cDNA template diluted 1:10 and MaximaTM SYBR Green/ROX qPCR Master Mix (2 ×) (Thermo Fisher Scientific, Waltham, MA, USA) using the 7500 Real Time PCR System (Applied Biosystems, Foster City, CA, USA). For each gene analyzed, a specific pair of primers was designed using the online Primer 3 program (www.fokker.wi.mit.edu/primer3/input.htm). The primer sequences are listed in Table 8. The amplification conditions started with initial denaturation at 95 °C for 10 min, followed by 40 cycles of 95 °C for 1 min and 60 °C for 1 min. The specificity of the product was assessed from the melting curve analysis. Average gene Ct values were normalized to the average *GAPDH* values of the same cDNA sample. Of note, the choice of experimental method did not appear to have any effect on *GAPDH* gene expression. No amplification was observed for RNA not subjected to reverse transcriptase during cDNA synthesis or in PCR reactions using water instead of a template. Gene expression levels were determined using the comparative Ct method.

### 4.7. Bioinformatics Analysis

To identify the statistically-significant biological functions and signaling pathways associated with differentially-expressed genes in leukocytes of the GDM/*SIRT1*(↑) pregnancies (i.e., *SIRT1*, *ACLY*, *G6PD*, *IL6*, and *SNAP23*, *p <* 0.05), Ingenuity Pathways Analysis was performed (IPA; Ingenuity H Systems, Redwood City, CA, USA; http://www.ingenuity.com). For this purpose, expression values of these genes obtained from RT-qPCR verification analysis were uploaded into the IPA Tool system. The core analysis was overlaid with the global molecular network in the Ingenuity Pathway Knowledge Base (IPKB) and highly interconnected gene networks and pathways were constructed. The significance of the associations between the data set and gene networks and canonical pathways was determined by a *p* value calculated using the Fisher’s exact test. *p* ≤ 0.05 was considered statistically significant.

### 4.8. Statistical Analysis

Data are presented as medians with interquartile ranges (25–75th percentile). The distribution of the analyzed biochemical and expression data was checked by the Shapiro-Wilk test. Differences of non-normally distributed variables between two groups were assessed using the non-parametric Mann-Whitney *U* (Wilcoxon’s) test. The Spearman’s rank correlation coefficients were calculated to evaluate univariate correlations between leukocyte *SIRT1* gene expression and clinical parameters of patients. Differences of each variable between three groups (i.e., NGT, GDM/*SIRT1*(↔), and GDM/*SIRT1*(↑) groups) were compared using the Kruskal-Wallis test. The pairwise post hoc test for multiple comparisons of mean rank of sums was employed to determine which specific groups differed from each other [74]. To evaluate leukocyte *SIRT1* expression between the GDM and NGT groups with adjustment for age and obesity variables (pre- and pregnancy BMI and gestational weight gain), ANCOVA analysis was performed. *p* value ≤0.05 was considered significant. Statistical analyses were performed using commercially-available statistics analysis software (Statistica version 8.0, StatSoft, Poland, license AXAP911E504325AR-K). 

## 5. Conclusions

In this study, an integrative approach combining PCR technology with bioinformatics was used to elucidate genetic factors associated with leukocyte *SIRT1* overexpression in GDM. To the best of our knowledge, this is the first study that provides novel and valuable data information about the relationship of leukocyte *SIRT1* expression with diabetic pregnancy at the time of GDM diagnosis. First, we found significantly elevated leukocyte *SIRT1* mRNA in a heterogeneous in regard to its expression group of hyperglycemic diabetic pregnancies, which correlated with glucose metabolism. It indicates that GDM affects alterations in *SIRT1* at the level of its gene expression and it process is linked to changes in glucose homeostasis. Second, we documented the differential transcriptional response of leukocytes to *SIRT1* over-abundance in GDM patients. Specifically, we found 11 genes with at least two-fold or more up-and down-regulated expression, which are engaged in biological processes implicated in the pathogenesis of T2DM. Hence, they might play a potentially important role in GDM-induced development of metabolic disturbances. Third, we confirmed a significant expression of four out of six genes identified by the PCR array approach in an extended GDM/*SIRT1*(↑) samples, indicating that moderate up-regulation of leukocyte *SIRT1* expression (three-fold) in the hyperglycemic GDM/*SIRT1*(↑) group works in concert with significant up-regulation (*IL6*, *G6PD*, and *SNAP23*) and down-regulation (*ACLY*) of diabetes-related genes involved in cell metabolism, inflammation, and molecular transport and trafficking. This is consistent with the conception that *SIRT1* overexpression can reflect an adaptive/protective response to expression alterations of the aforementioned genes in leukocytes. Fourth, by using gene network and pathways analyses in *SIRT1*, *IL6*, *G6PD*, *SNAP23*, and *ACLY* genes, we revealed a potential role of several important biological processes and pathways mainly related to cellular and immunological processes, which may be engaged in changes of metabolic phenotypes of the GDM/*SIRT1*(↑) patients. However, further investigations in the future will be imperative in clarifying the functional and biological importance of these pathways in GDM. Fifth, by comparing leukocyte gene expression of the aforementioned four genes between the GDM/*SIRT1*(↑) and GDM/*SIRT1*(↔) groups, we identified a unique *ACLY* under-expression in GDM/*SIRT1*(↑) women, suggesting a biomarker candidate for further study. Of note, while it is not yet known how *SIRT1* over-abundance might affect the decreased *ACLY* expression in hyperglycemic GDM patients, these results, though still exploratory, may represent a first step towards elucidating the beneficial influence of SIRT1 on ACL regulation under diabetic conditions. Hence, further investigations with the use of diabetic disease models are needed to clarify the underlying molecular mechanism of this effect. From a long-term perspective, this knowledge could help develop more effective therapies against diabetes.

Taken together, these findings are particularly important, as they indicate the relevance of diabetes-related gene expression pattern and pathways related to leukocyte *SIRT1* overexpression in GDM and thereby provide insight into the transcriptomics of leukocytes in diabetic pregnancy. Although the expression pattern directs attention to cell metabolism, inflammation, and molecular transport and trafficking, functional validation of the identified genes is needed to establish their potential role in the development of GDM.

Given the increasing prevalence of T2DM in women with a history of GDM, there is an urgent need to understand the molecular mechanisms underlying this phenomenon. In this context, intensive research on temporal quantitative leukocyte *SIRT1* gene expression changes during pregnancy and the postpartum period after pregnancy complicated by GDM is currently under way in our group.

## Figures and Tables

**Table 1:** Clinical and leukocyte *SIRT1* mRNA expression data for different experimental groups.

Variable	NGT (*n* = 41)	GDM (*n* = 122)	*p #*	GDM/*SIRT1*(↔) (*n* = 92)	GDM/*SIRT1*(↑) (*n* = 30)	*p ##*
Age [years]	29.0 (26.0–33.0)	30.5 (27.0–33.0)	0.156	31.0 (27.5–33.0)	29.5 (26.0–33.0)	0.265
Pre-pregnancy BMI [kg/m^2^]	24.2 (21.4–26.6)	24.3 (21.1–27.7)	0.648	24.0 (21.1–27.7)	24.6 (21.9–27.7)	0.845
Pregnancy BMI [kg/m^2^]	28.0 (25.0–30.5)	27.5 (24.5–32.1)	0.905	27.5 (24.2–32.1)	28.1 (25.0–32.7)	0.932
Gestational weight gain [kg]	10.0 (6.0–12.0)	8.2 (6.0–12.0)	0.244	8.0 (6.0–11.8)	8.9 (6.0–13.0)	0.500
TGs [mg/dL]	214.5 (179.0–277.1)	217.9 (175.5–259.0)	0.477	217.9 (174.0–259.0)	217.8 (175.5–264.0)	0.772
TC [mg/dL]	261.3 (238.5–282.5)	251.1 (226.0–282.3)	0.303	254.0 (226.0–283.0)	249.7 (219.0–275.0)	0.570
HDL-C [mg/dL]	76.3 (60.6–85.5)	70.5 (59.3–82.5)	0.810	74.0 (61.0–84.0)	65.0 (53.0–73.0)	0.279
LDL-C [mg/dL]	141.5 (123.5–168.5)	136.0 (114.0–158.0)	0.547	134.5 (114.0–158.0)	145.0 (133.0–154.0)	0.571
HbA1c [%]	5.3 (5.0–5.6)	5.4 (5.2–5.7)	0.090	5.4 (5.2–5.7)	5.4 (5.2–5.7)	0.223
FPG [mg/dL]	81.0 (74.5–84.0)	88.0 (80.0–97.0)	0.000	88.0 (79.5–97.5)	92.0 (80.0–97.0)	0.000 ^A,B^
1-h OGTT [mg/dL]	164.5 (126.0–185.0)	183.0 (168.0–202.0)	0.000	182.0 (168.0–202.0)	189.0 (160.0–200.0)	0.000 ^A,B^
2-h OGTT [mg/dL]	122.0 (102.0–132.0)	157.0 (148.0–176.0)	0.000	156.0 (147.0–173.5)	158.0 (152.0–178.0)	0.000 ^A,B^
CRP [mg/L]	3.9 (2.1–8.3)	3.3 (2.0–5.9)	0.308	3.4 (2.0–6.3)	3.1 (2.3–5.8)	0.591
Insulin [µlU/mL]	7.1 (1.7–10.1)	5.2 (2.8–8.5)	0.602	5.2 (2.9–8.8)	5.2 (2.4–8.5)	0.790
HOMA-IR	1.3 (0.5–2.2)	1.2 (0.6–1.9)	0.972	1.2 (0.6–1.9)	1.3 (0.6–1.9)	0.995
*SIRT1*	0.59 (0.00–1.00)	0.78 (0.27–1.41)	0.041	0.56 (0.16–0.89)	1.81 (1.54–2.28)	0.000 ^B,C^

Abbreviations: BMI, body mass index; CRP, C reactive protein; FPG, fasting plasma glucose; HDL, high-density lipoprotein; HOMA-IR, homeostasis model assessment of insulin resistance; LDL, low density lipoprotein; TC, total cholesterol; TGs, triglycerides. Data are presented as median and interquartile range (25–75 percentiles). *# p* values assessed by Wilcoxon’s test; *## p* values between NGT, GDM/*SIRT1*(↔), and GDM/*SIRT1*(↑) groups assessed by Kruskal-Wallis’ test; *p <* 0.05 GDM/*SIRT1*(↔) vs. NGT groups (A), GDM/*SIRT1*(↑) vs. NGT groups (B), and GDM/*SIRT1*(↔) vs. GDM/*SIRT1*(↑) groups (C) as assessed by post hoc pairwise multiple comparisons of mean rank sums.

**Table 2:** Spearman correlation analysis of leukocyte *SIRT1* gene expression with clinical parameters in the entire study groups.

Variable	NGT+GDM (*n* = 163)		NGT+GDM/*SIRT1*(↔) (*n* = 133)		NGT+GDM/*SIRT1*(↑) (*n* = 71)	
	*R* (95% CI)	*p*	*R* (95% CI)	*p*	*R* (95%CI)	*p*
Age [years]	−0.03 (−0.18, 0.13)	0.721	0.01 (−0.16, 0.18)	0.938	−0.02 (−0.25, 0.22)	0.888
Pre−pregnancy BMI [kg/m^2^]	0.10 (−0.05, 0.25)	0.197	0.12 (−0.06, 0.28)	0.187	0.12 (−0.11, 0.35)	0.325
Pregnancy BMI [kg/m^2^]	0.04 (−0.11, 0.20)	0.581	0.04 (−0.14, 0.21)	0.679	0.07 (−0.17, 0.30)	0.553
Gestational weight gain [kg]	−0.12 (−0.27, 0.03)	0.127	−0.16 (−0.32, 0.02)	0.069	−0.12 (−0.34, 0.12)	0.318
TC [mg/dL]	−0.01 (−0.20, 0.19)	0.919	0.03 (−0.19, 0.24)	0.815	−0.02 (−0.29, 0.26)	0.911
TGs [mg/dL]	0.06 (−0.14, 0.25)	0.575	0.09 (−0.12, 0.30)	0.401	0.06 (−0.22, 0.33)	0.695
HDL-C [mg/dL]	−0.17 (−0.36, 0.02)	0.080	−0.10 (−0.31, 0.11)	0.345	−0.25 (−0.49, 0.03)	0.082
LDL-C [mg/dL]	−0.00 (−0.20, 0.19)	0.969	−0.08 (−0.29, 0.14)	0.474	0.01 (−0.27, 0.29)	0.942
HbA1c [%]	0.04 (−0.12, 0.20)	0.616	0.03 (−0.15, 0.20)	0.774	0.31 (0.08, 0.51)	0.010 *
FPG [mg/dL]	0.07 (−0.09, 0.22)	0.406	−0.03 (−0.21, 0.15)	0.733	0.41 (0.19, 0.59)	0.000 *
1-h OGTT [mg/dL]	−0.00 (−0.17, 0.17)	0.993	−0.05 (−0.24, 0.14)	0.615	0.32 (0.06, 0.53)	0.015 *
2-h OGTT [mg/dL]	0.23 (0.07, 0.37)	0.005 *	0.08 (−0.09, 0.25)	0.368	0.76 (0.64, 0.85)	0.000 *
CRP [mg/L]	−0.01 (−0.18, 0.16)	0.922	0.04 (−0.15, 0.22)	0.702	−0.01 (−0.26, 0.24)	0.924
Insulin [µlU/mL]	−0.13 (−0.29, 0.04)	0.138	−0.12 (−0.30, 0.07)	0.196	−0.15 (−0.38, 0.10)	0.233
HOMA-IR	−0.08 (−0.25, 0.10)	0.386	−0.11 (−0.30, 0.09)	0.285	−0.06 (−0.31, 0.19)	0.623

BMI, body mass index; CRP, C-reactive protein; FPG, fasting blood glucose; HDL, high-density lipoprotein; HOMA-IR, homeostasis model assessment of insulin resistance; LDL, low-density lipoprotein; TC, total cholesterol; TGs, triglycerides. * *p* < 0.05 as assessed by Spearman’s rank order correlation analysis; 95% confidence interval (CI) for correlation coefficient.

**Table 3:** ANCOVA analysis of leukocyte *SIRT1* expression between the GDM and NGT groups (Status) with adjustment for age and obesity variables (pre- and pregnancy BMI and gestational weight gain).

Variable	*DF*	*MS*	*F*	*p*
Age	1	0.34	0.74	0.392
Pre-pregnancy BMI	1	0.11	0.24	0.627
Pregnancy BMI	1	0.15	0.32	0.574
Gestational weight gain	1	0.29	0.62	0.431
Status	1	2.17	4.72	0.031

*DF*, degrees of freedom; *F*, Fisher’s test; *MS*, mean square; *p <* 0.05 for analysis of covariance (ANCOVA) between two groups with adjustment for the corresponding variables.

**Table 4:** List of 11 diabetes-related genes with at least ± 2-fold regulation in leukocytes from the GDM/*SIRT1*(↑) pregnancies (*n* = 9) compared to the NGT controls (*n* = 7).

Unigene	GenBank		Symbol	Description	FC
				Receptors, Transporters & Channels *	
Hs,431279	NM_006178		*NSF*	*N*-ethylmaleimide-sensitive factor	0.48
Hs,511149	NM_003825		*SNAP23*	Synaptosomal-associated protein, 23 kDa	3.90
Hs,515104	NM_006949		*STXBP2*	Syntaxin binding protein 2	0.50
				**Metabolic Enzymes**	
Hs,387567	NM_001096		*ACLY*	ATP citrate lyase	0.48
Hs,461047	NM_000402		*G6PD*	Glucose-6-phosphate dehydrogenase	2.81
Hs,524418	NM_005276		*GPD1*	Glycerol-3-phosphate dehydrogenase 1 (soluble)	0.49
				**Cytokines & Growth Factors**	
Hs,654458	NM_000600		*IL6*	Interleukin 6 (interferon, beta 2)	2.07
				**Signal Transduction**	
Hs,471508	NM_005544		*IRS1*	Insulin receptor substrate 1	0.49
Hs,442344	NM_003749		*IRS2*	Insulin receptor substrate 2	2.31
				**Transcription Factors**	
Hs,32938	NM_000209		*PDX1*	Pancreatic and duodenal homeobox 1	0.33
Hs,592123	NM_004176		*SREBF1*	Sterol regulatory element binding transcription factor 1	0.32

* Grouped according to function based on Qiagen listing. The fold changes (FC) are listed for each gene.

**Table 5:** Comparison of differences in gene expression between GDM and NGT for each of the six genes, as indicated by PCR array and RT-qPCR verification.

*Symbol*	*Description*	*PCR Array*	*RT-qPCR Verification*	
		*GDM/SIRT1*(↑) vs. *NGT*	*GDM/SIRT1*(↑) vs. *NGT*	*GDM/SIRT1*(↔) vs. *NGT*
	***Receptors, Transporters & Channels ^§^***			
*SNAP23*	Synaptosomal-associated protein, 23 kDa	3.90	6.55 *	3.93
	***Metabolic enzyme***			
*ACLY*	ATP citrate lyase	0.48	0.63 *	1.11
*G6PD*	Glucose-6-phosphate dehydrogenase	2.81	1.52 *	1.06
	***Cytokines & Growth Factors***			
*IL6*	Interleukin 6 (interferon, beta 2)	2.07	2.28 *	1.88 *
	***Signal Transduction***			
*IRS2*	Insulin receptor substrate 2	2.31	2.62	1.69
	***Transcription Factors***			
*SREBF1*	Sterol regulatory element binding transcription factor 1	0.32	0.33	0.68

**^§^** Grouped according to function based on Qiagen listing. * *p* < 0.05 as assessed by Kruskal-Wallis test followed by post hoc pairwise multiple comparisons. The fold changes (FC) are listed for each gene.

**Table 6:** Biological function associated with GDM/*SIRT1*(↑) identified based on Ingenuity Pathway Analysis (IPA).

Category	Top Function	*p* Range	Number of Targets
**Disease and disorder**			
1	Inflammatory response	6.60 × 10^−6^–3.24 × 10^−2^	3
2	Cardiovascular disease	2.54 × 10^−5^–4.55 × 10^−2^	3
3	Organismal injury and abnormalities	2.54 × 10^−5^–4.87 × 10^−2^	4
4	Immunological disease	9.57 × 10^−5^–2.33 × 10^−2^	2
5	Inflammatory disease	9.57 × 10^−5^−1.53 × 10^−2^	2
**Molecular and cellular function**			
1	Cell-to cell signaling and interaction	6.60 × 10^−6^–3.15 × 10^−2^	2
2	Cellular development	2.10 × 10^−5^–4.70 × 10^−2^	4
3	Drug metabolism	3.82 × 10^−5^–1.41 × 10^−2^	2
4	Molecular transport	3.82 × 10^−5^–3.04 × 10^−2^	5
5	Small molecule biochemistry	3.82 × 10^−5^–3.04 × 10^−2^	4
**Physiological system development and function**			
1	Hematological system development and function	6.60 × 10^−6^–4.82 × 10^−2^	4
2	Immune cell trafficking	6.60 × 10^−6^–3.24 × 10^−2^	2
3	Cardiovascular system development and function	2.54 × 10^−5^–4.44 × 10^−2^	3
4	Organ morphology	2.54 × 10^−5^–4.44 × 10^−2^	3
5	Organismal development	2.54 × 10^−5^–4.23 × 10^−2^	3

This selection is organized by the negative logarithm of *p*-values (Fisher test), calculated by IPA ([-Log (0.05) = 1.3]). The *p*-value range indicates the *p*-values of the various pathways and processes belonging to that category. The number of targets indicates the total number of genes associated with the functional category.

**Table 7:** List of top five most significant canonical pathways.

Pathways	*p*
Sirtuin signaling pathway	4.20 × 10^−5^
Acetyl-CoA biosynthesis III (from citrate)	3.01 × 10^−4^
Pentose phosphate pathway (oxidative branch)	1.20 × 10^−3^
Pentose phosphate pathway	3.01 × 10^−3^
Differential regulation of cytokine production in macrophage and T helper cells by IL-17A and IL-17F	5.41 × 10^−3^

**Table 8:** Primer sequences used for PCR reactions.

Gene Symbol	Primer Sequence 5’→3’	Amplicon Size (bp)
*Semi-quantitative PCR*		
*SIRT1*	F: TCACCACCAGATTCTTCAGTG R: CCTCTTGATCATCTCCATCAGTC	544
*GAPDH*	F: CTGCACCACCAACTGCTTAG R: GTTGCTGTAGCCAAATTCGTTG	514
*RT-qPCR*		
*ACLY*	F: TCTTTGTGCTGGGAAGGAGT R: GTAGGGTGCCTACTGCTATG	90
*G6PD*	F: ACGTCCGTGATGAGAAGGTC R: GTGGGGTCGTCCAGGTAC	133
*IL6*	F: CCTGAGAAAGGAGACATGTAACAAG R: AAGGTTCAGGTTGGTTTTCTGCC	79
*IRS2*	F: CGGTGAGTTCTACGGGTACAT R: TCAGGGTGTATTCATCCAGCG	194
*SNAP23*	F: TAGCCATTGAGTCTCAGGATG R: GGTTTAGTTGTTCCTTTTGTTCA	72
*SREBF1*	F: ACAGTGACTTCCCTGGCCTAT R: GCATGGACGGGTACATCTTCAA	222
*GAPDH*	F: GGTGGTCTCCTCTGACTTCAACA R: GTTGCTGTAGCCAAATTCGTTGT	27

F, forward; R, reverse.

## Supplementary Materials

Supplementary materials can be found at http://www.mdpi.com/1422-0067/19/12/3826/s1.

## Abbreviations

ACL	ATP citrate lyase
COX2	Cyclooxygenase 2
ECFC	Endothelial colony-forming cell
GDM	Gestational diabetes mellitus
G6PD	Glucose-6-phosphate dehydrogenase
HFD	High fat diet
HUVEC	Human umbilical vein endothelial cells
IL6	Interleukin 6
IPA	Ingenuity Pathway Analysis
MAPK	Mitogen-activated protein kinases
NGT	Normal glucose tolerance
SIRT1	Sirtuin 1
SNAP23	Synaptosomal-associated protein 23
STACs	Sirtuin-activating compounds
T2DM	Type 2 diabetes mellitus
