# Supplementary material for: Expression Profile of Diabetes-Related Genes Associated with Leukocyte Sirtuin 1 Overexpression in Gestational Diabetes
Source: Int J Mol Sci. 2018 Nov 30;19(12):3826. doi: 10.3390/ijms19123826 (PMC6321739; doi:10.3390/ijms19123826)
Supplement: Supplementary file 1 [file ijms-19-03826-s001.pdf]

Table S1. Complete gene expression data from Human Diabetes RT² Profiler™ PCR Array

Position	Unigene	GenBank	Symbol	Description	FC	Average Ct	
				**Receptors, Transporters & Channels***		NGT	GDM
A01	Hs,5447	NM_000352	*ABCC8*	ATP-binding cassette, sub-family C (CFTR/MRP), member 8	0.93	35.00	35.00
A04	Hs,2549	NM_000025	*ADRB3*	Adrenergic, beta-3-, receptor	0.88	34.92	35.00
A07	Hs,1307	NM_000486	*AQP2*	Aquaporin 2 (collecting duct)	0.93	35.00	35.00
A09	Hs,5117	NM_001123	*CCR2*	Chemokine (C-C motif) receptor 2	1.00	26.28	26.17
A10	Hs,5916	NM_006139	*CD28*	CD28 molecule	0.81	34.51	34.71
A11	Hs,5126	NM_001712	*CEACAM1*	Carcinoembryonic antigen-related cell adhesion molecule 1	1.31	26.08	25.59
B01	Hs,2478	NM_005214	*CTLA4*	Cytotoxic T-lymphocyte-associated protein 4	0.53	30.90	31.71
B11	Hs,208	NM_000160	*GCGR*	Glucagon receptor	0.93	35.00	35.00
C01	Hs,3891	NM_002062	*GLP1R*	Glucagon-like peptide 1 receptor	0.93	35.00	35.00
C07	Hs,6434	NM_000201	*ICAM1*	Intercellular adhesion molecule 1	0.84	28.74	28.89
D02	Hs,5134	NM_000418	*IL4R*	Interleukin 4 receptor	0.64	34.22	34.75
D06	Hs,4657	NM_000208	*INSR*	Insulin receptor	0.93	35.00	35.00
E05	Hs,4312	NM_006178	*NSF*	N-ethylmaleimide-sensitive factor	**0.48**	28.42	29.37
F08	Hs,2961	NM_004578	*RAB4A*	RAB4A, member RAS oncogene family	0.88	20.55	20.63
F10	Hs,7287	NM_000655	*SELL*	Selectin L	0.97	23.89	23.83
F11	Hs,3806	NM_001042	*SLC2A4*	Solute carrier family 2 (facilitated glucose transporter), member 4	0.77	34.72	35.00
F12	Hs,5111	NM_003825	***SNAP23***	Synaptosomal-associated protein, 23kDa	**3.90**	30.55	28.49
G01	Hs,1673	NM_003081	*SNAP25*	Synaptosomal-associated protein, 25kDa	0.62	25.77	26.35
G03	Hs,8373	NM_004604	*STX4*	Syntaxin 4	0.59	24.88	25.52
G04	Hs,2882	NM_003165	*STXBP1*	Syntaxin binding protein 1	0.93	35.00	35.00
G05	Hs,5151	NM_006949	*STXBP2*	Syntaxin binding protein 2	**0.50**	22.44	23.33
G08	Hs,2795	NM_001065	*TNFRSF1A*	Tumor necrosis factor receptor superfamily, member 1A	1.23	22.57	22.17
G10	Hs,6670	NM_004781	*VAMP3*	Vesicle-associated membrane protein 3 (cellubrevin)	0.77	24.80	25.07
G11	Hs,6999	NM_194434	*VAPA*	VAMP (vesicle-associated membrane protein)-associated protein	1.16	22.97	22.65
				**Nuclear Receptors**			
E11	Hs,1031	NM_005036	*PPARA*	Peroxisome proliferator-activated receptor alpha	0.84	34.26	34.41

E12	Hs,1626	NM_015869	*PPARG*	Peroxisome proliferator-activated receptor gamma	0.68	30.63	31.09
				**Metabolic Enzymes**			
A02	Hs,6544	NM_000789	*ACE*	Angiotensin I converting enzyme (peptidyl-dipeptidase A) 1	0.86	34.74	34.85
A03	Hs,3875	NM_001096	***ACLY***	ATP citrate lyase	**0.48**	26.27	27.24
B03	Hs,5272	NM_006208	*ENPP1*	Ectonucleotidepyrophosphatase/phosphodiesterase 1	1.36	34.79	34.24
B04	Hs,4944	NM_000507	*FBP1*	Fructose-1,6-bisphosphatase 1	1.44	26.82	26.19
B08	Hs,2122	NM_000151	*G6PC*	Glucose-6-phosphatase, catalytic subunit	0.93	35.00	35.00
B09	Hs,4610	NM_000402	***G6PD***	Glucose-6-phosphate dehydrogenase	**2.80**	27.51	25.92
B12	Hs,1270	NM_000162	*GCK*	Glucokinase (hexokinase 4)	0.93	35.00	35.00
C02	Hs,5244	NM_005276	*GPD1*	Glycerol-3-phosphate dehydrogenase 1 (soluble)	**0.49**	33.75	34.68
C03	Hs,4457	NM_002093	*GSK3B*	Glycogen synthase kinase 3 beta	1.01	25.20	25.08
C04	Hs,5175	NM_002133	*HMOX1*	Hemeoxygenase (decycling) 1	1.08	26.34	26.12
C08	Hs,5005	NM_004969	*IDE*	Insulin-degrading enzyme	1.07	29.93	29.73
D11	Hs,2116	NM_002395	*ME1*	Malic enzyme 1, NADP(+)-dependent, cytosolic	0.51	30.82	31.69
E03	Hs,7079	NM_000603	*NOS3*	Nitric oxide synthase 3 (endothelial cell)	0.64	28.44	28.98
E06	Hs,1777	NM_001618	*PARP1*	Poly (ADP-ribose) polymerase 1	0.54	29.01	29.79
F03	Hs,4332	NM_006251	*PRKAA1*	Protein kinase, AMP-activated, alpha 1 catalytic subunit	1.21	23.84	23.47
F04	Hs,6470	NM_016203	*PRKAG2*	Protein kinase, AMP-activated, gamma 2 non-catalytic subunit	0.98	26.64	26.56
F05	Hs,4603	NM_002738	*PRKCB*	Protein kinase C, beta	1.14	22.65	22.36
F07	Hs,2824	NM_002863	*PYGL*	Phosphorylase, glycogen, liver	0.80	26.29	26.52
				**Cytokines & Growth Factors**			
A05	Hs,1938	NM_000029	*AGT*	Angiotensinogen (serpin peptidase inhibitor, clade A, member 8)	0.93	35.00	35.00
A08	Hs,5148	NM_002985	*CCL5*	Chemokine (C-C motif) ligand 5	0.70	24.35	24.76
B10	Hs,5164	NM_002054	*GCG*	Glucagon	0.93	35.00	35.00
C09	Hs,856	NM_000619	*IFNG*	Interferon, gamma	0.58	33.08	33.76
C12	Hs,1937	NM_000572	*IL10*	Interleukin 10	0.87	34.62	34.72
D01	Hs,674	NM_002187	*IL12B*	Interleukin 12B (natural killer cell stimulatory factor 2, cytotoxic	0.73	22.30	22.65
D03	Hs,6544	NM_000600	***IL6***	Interleukin 6 (interferon, beta 2)	**2.07**	28.65	27.50
D05	Hs,6545	NM_000207	*INS*	Insulin	1.15	30.67	30.36
F09	Hs,2830	NM_020415	*RETN*	Resistin	0.93	35.00	35.00
G06	Hs,6452	NM_000660	*TGFB1*	Transforming growth factor, beta 1	0.93	35.00	35.00
G07	Hs,2415	NM_000594	*TNF*	Tumor necrosis factor	1.40	27.30	26.71
G12	Hs,7379	NM_003376	*VEGFA*	Vascular endothelial growth factor A	0.62	28.78	29.37

				**Signal Transduction**			
A06	Hs,6315	NM_001626	*AKT2*	V-akt murine thymoma viral oncogene homolog 2	1.01	28.67	28.56
B02	Hs,4179	NM_057158	*DUSP4*	Dual specificity phosphatase 4	0.58	26.62	27.31
C10	Hs,6072	NM_000599	*IGFBP5*	Insulin-like growth factor binding protein 5	0.90	26.62	26.66
C11	Hs,5976	NM_001556	*IKBKB*	Inhibitor of kappa light polypeptide gene enhancer in B-cells,	1.46	31.66	31.01
D04	Hs,5238	NM_001567	*INPPL1*	Inositol polyphosphate phosphatase-like 1	0.93	35.00	35.00
D07	Hs,4715	NM_005544	*IRS1*	Insulin receptor substrate 1	**0.49**	31.75	32.66
D08	Hs,4423	NM_003749	***IRS2***	Insulin receptor substrate 2	**2.31**	27.22	25.91
D09	Hs,4852	NM_001315	*MAPK14*	Mitogen-activated protein kinase 14	0.91	23.49	23.51
D10	Hs,1382	NM_002750	*MAPK8*	Mitogen-activated protein kinase 8	0.78	27.00	27.26
E08	Hs,4974	NM_002646	*PIK3C2B*	Phosphoinositide-3-kinase, class 2, beta polypeptide	0.93	26.35	26.36
E09	Hs,5184	NM_005026	*PIK3CD*	Phosphoinositide-3-kinase, catalytic, delta polypeptide	0.84	29.00	29.15
E10	Hs,1322	NM_181504	*PIK3R1*	Phosphoinositide-3-kinase, regulatory subunit 1 (alpha)	1.62	34.63	33.82
F06	Hs,4175	NM_002827	*PTPN1*	Protein tyrosine phosphatase, non-receptor type 1	1.04	26.83	26.67
G09	Hs,5168	NM_021158	*TRIB3*	Tribbles homolog 3 (Drosophila)	0.99	30.40	30.31
				**Transcription Factors**			
A12	Hs,6994	NM_004364	*CEBPA*	CCAAT/enhancer binding protein (C/EBP), alpha	0.97	25.70	25.65
B05	Hs,4364	NM_005251	*FOXC2*	Forkhead box C2 (MFH-1, mesenchyme forkhead 1)	0.93	35.00	35.00
B06	Hs,6959	NM_005249	*FOXG1*	Forkhead box G1	0.93	35.00	35.00
B07	Hs,2477	NM_014009	*FOXP3*	Forkhead box P3	0.63	32.70	33.25
C05	Hs,1911	NM_000458	*HNF1B*	HNF1 homeobox B	0.66	33.05	33.55
C06	Hs,1164	NM_178849	*HNF4A*	Hepatocyte nuclear factor 4, alpha	0.77	25.22	25.49
D12	Hs,5746	NM_002500	*NEUROD1*	Neurogenic differentiation 1	0.93	35.00	35.00
E01	Hs,6544	NM_003998	*NFKB1*	Nuclear factor of kappa light polypeptide gene enhancer in B-cells	1.25	24.24	23.81
E02	Hs,7053	NM_003317	*NKX2-1*	NK2 homeobox 1	0.85	31.71	31.83
E04	Hs,6543	NM_005011	*NRF1*	Nuclear respiratory factor 1	0.69	26.19	26.62
E07	Hs,3293	NM_000209	*PDX1*	Pancreatic and duodenal homeobox 1	**0.33**	23.83	25.32
F01	Hs,5270	NM_013261	*PPARGC1A*	Peroxisome proliferator-activated receptor gamma, coactivator 1	1.02	27.15	27.01
F02	Hs,5912	NM_133263	*PPARGC1B*	Peroxisome proliferator-activated receptor gamma, coactivator 1	1.75	27.91	27.00
G02	Hs,5921	NM_004176	***SREBF1***	Sterol regulatory element binding transcription factor 1	**0.32**	31.37	32.93
				**House-Keeping Genes – Endogenous Controls**			
H01	Hs,5206	NM_001101	*ACTB*	Actin, beta	0.95	18.07	18.04
H02	Hs,5342	NM_004048	*B2M*	Beta-2-microglobulin	1.20	28.15	27.78

H03	Hs,5923	NM 002046	*GAPDH*	Glyceraldehyde-3-phosphate dehydrogenase	0.92	22.99	23.02
H04	Hs,4127	NM 000194	*HPRT1*	Hypoxanthine phosphoribosyltransferase 1	0.79	21.07	21.30
H05	Hs,5462	NM 001002	*RPLP0*	Ribosomal protein, large, P0	1.20	18.20	17.83

*Grouped according to function based on Qiagen listing; The fold changes (FC) are listed for each gene.
